# The latent profile structure of negative emotion in female college students and its impact on eating behavior: the mediating role of physical exercise

**DOI:** 10.3389/fpubh.2025.1663474

**Published:** 2025-08-13

**Authors:** Wenying Huang, Bin Chen, Chang Hu

**Affiliations:** Physical Education College, Jiangxi Normal University, Nanchang, China

**Keywords:** negative emotion, eating behavior, physical exercise, female college students, moderated mediation model, latent profile analysis

## Abstract

**Objective:**

Negative emotions (NE) are strongly linked to disordered eating among female university students, yet the underlying mechanisms require further clarification. This study aimed to investigate the relationship between NE and eating behavior (EB) within this population, specifically examining the mediating role of physical exercise (PE) and exploring the latent profiles of NE through a person-centered approach.

**Methods:**

An online survey was administered to 832 female university students, from which 789 valid responses were analyzed. We utilized the Depression Anxiety Stress Scale (DASS), the Sakata Eating Behavior Scale short form (EBS-SF), and a single-item PE measure. The data were analyzed using mediation analysis with bootstrapping and Latent Profile Analysis (LPA).

**Results:**

NE was significantly and positively related to problematic EB (*β* = 0.414, *p* < 0.001) and negatively related to PE (*β* = −0.390, *p* < 0.001), while PE was negatively related to EB (*β* = −0.086, *p* < 0.05). PE was confirmed as a partial mediator in the NE-EB relationship (indirect effect = 0.034). Furthermore, LPA identified three distinct NE profiles: an “Emotionally Stable” group (16.86%), a “Transition Risk” group (51.33%), and an “Anxious-Distress” group (31.81%), with these profiles exhibiting significant differences in both EB (*F* = 65.088) and PE (*F* = 55.241).

**Conclusion:**

Female university students can be classified into three distinct emotional profiles (“Emotionally Stable,” “Transition Risk,” and “Anxious-Distress”), which are significantly associated with different levels of physical exercise and problematic eating behaviors. In addition, NE is not only directly associated with EB but also indirectly linked to it by reducing engagement in PE. This finding indicates that health interventions targeting female college students with high NE should, in addition to emotional management, actively promote the development of PE habits to effectively block the pathway from NE to unhealthy eating behaviors. The conclusions should be considered in light of the study’s cross-sectional design and its reliance on a single-item measure for PE.

## 1 Introduction

Nutrition is widely recognized as the cornerstone of individual health (1–3). However, with global economic development and shifts in lifestyle patterns, overnutrition has become a prominent global public health concern (4–7). According to a 2022 report by the World Health Organization, the global population of overweight or obese individuals has surpassed 1 billion (8, 9), with the prevalence of obesity among young women also exhibiting an upward trend (10–12). Unhealthy eating behaviors (UEB) have been identified as a primary risk factor for non-communicable chronic diseases (cardiovascular disease, type 2 diabetes) (13–15). Statistical evidence indicates that over 30% of global deaths annually are attributable to these diseases (16, 17).

In this context, eating behaviors (EB) have garnered significant academic attention due to their role as a pivotal factor influencing individual nutrient intake and physical health (18–22). When considering various demographic groups, the EB of female university students warrants particular attention (23–25). Large-scale epidemiological studies have confirmed that eating disorders are on the rise among young women worldwide (26). A meta-analysis of college students clearly indicates that the prevalence of UEB among female college students is significantly higher than in other groups, making them a key population for studying EB and its influencing factors (27). Recent studies have further uncovered the underlying mechanisms behind this phenomenon. On one hand, the pervasive influence of social media has intensified body image anxiety and social comparison among this demographic (28–31). On the other hand, academic and interpersonal pressures often drive them to seek comfort through emotional eating (32–34). These findings collectively suggest that UEB among female university students is not only widespread but also rooted in complex psychological mechanisms that warrant in-depth investigation.

Among the numerous psychological factors influencing the EB of female college students, negative emotions (NE) undoubtedly play a central role (35–38). However, previous studies examining this relationship have primarily adopted a “variable-centered approach (39, 40).” Firstly, this approach may overlook the heterogeneity of different NE coexisting within an individual (41–43). Secondly, because NE are seldom experienced in isolation but rather manifest as complex patterns of co-occurring feelings (i.e., heterogeneity) (44, 45), aggregating them into a single “total score” risks obfuscating the distinct influence pathways that different emotional profiles may have on EB. To address these limitations, this study will adopt a “person-centered “Latent Profile Analysis (LPA) method (46). This methodological framework enables us to address a fundamental question from a more holistic and nuanced perspective. Which specific combinations of NE put female college students at risk for eating disorders?

## 2 Literature review and hypotheses development

### 2.1 Negative emotions and eating behaviors among female college students

Negative emotions are generally characterized as adverse psychological experiences that individuals encounter when coping with stress or adverse life events (47, 48). These emotions encompass a wide spectrum, including depression, anxiety, anger, and repression (49). NE has been shown to have a significant association with mental health (50, 51), and is also closely associated with a range of maladaptive behaviors (52, 53). Affect Regulation Theory posits that when individuals encounter aversive emotions such as anxiety, depression, or stress, they may engage in maladaptive coping strategies to attain transient psychological relief. Binge eating is a typical manifestation of such maladaptive coping (54). In recent years, a large number of interdisciplinary studies have provided empirical support for this theory. For instance, a study involving 5,200 adult subjects demonstrated that for each one standard deviation increase in negative emotion intensity, the frequency of emotional eating increased by 23% (*p* < 0.01). This association was found to be significantly strengthened in individuals with weaker affect regulation abilities (55). Subsequent neuroimaging studies have identified that aberrant coupling between the limbic system, which is activated by NE, and the reward circuit significantly amplifies the appeal of high-calorie foods. This finding provides a physiological mechanism to elucidate the neurobiological basis of emotion-driven EB (56). Therefore, this study, grounded in emotion regulation theory, employed the LPA method to identify latent profile classifications of NE among female college students and examined differences in EB across these categories.

### 2.2 The mediating role of physical exercise

Physical exercise (PE) has been identified as a positive emotion regulation strategy that can modulate emotional states through both physiological (57, 58) and psychological (59, 60) mechanisms. According to the tenets of cognitive-behavioral theory (61), human cognition, emotion, and behavior are interconnected. Negative cognitive processes have the capacity to lead to NE (e.g., depression), which, in turn, can result in maladaptive behaviors (62). This dynamic perpetuates a vicious cycle. PE has been demonstrated to effectively disrupt the persistent cycle of NE by diverting attention (63, 64) and enhancing self-efficacy (65), thereby reducing UEB such as binge eating (66, 67), which are often caused by emotional dysregulation (68). Studies on the relationship between NE and PE have demonstrated that individuals who experience elevated levels of NE are more prone to enter into a negative cycle, which affects their PE levels (69). At the same time, PE has been demonstrated to directly alleviate NE, such as anxiety and depression (70). The mechanisms by which this occurs include the promotion of endorphin release and the reduction of cortisol levels (71). Alternatively, PE can enhance an individual’s psychological resilience (72), thereby increasing an individual’s ability to resist NE. Research on the association between PE and EB has demonstrated a significant positive correlation between these variables (73, 74). Specifically, the probability of engaging in UEB has been found to decrease as PE levels increase. Therefore, this study hypothesizes that PE may serve as an important potential mediator in the relationship between NE and EB.

### 2.3 The present study

In summary, this study aims to examine whether PE mediates the relationship between NE and EB, and to explore the different latent profiles of NE among female college students and their relationship with EB through LPA. This approach extends previous findings and provides a comprehensive perspective on the link between NE and EB. In light of the aforementioned theoretical perspectives and empirical research, the following hypotheses are proposed: (H1) PE mediates the relationship between NE and EB; (H2) NE exhibits different latent profiles among female college students; (H3) Different latent profiles of NE differ in their effects on EB.

## 3 Materials and methods

### 3.1 Participants and procedure

We conducted an *a priori* power analysis using G*Power 3.1, applying a standard regression-based mediation model. Assuming a medium effect size (f^2^ = 0.15), a significance level (*α* = 0.05), and power (1 − *β*) of 0.95 (75), the analysis indicated that a minimum of 119 participants was required. In addition, following conventional guidelines in psychological and behavioral research, the recommended sample size should be at least 10 times the number of questionnaire items (76). Given that our instrument included 29 items, the minimum sample size based on this criterion was 290 participants. Taking both considerations into account, we adopted 290 as the final minimum sample size for this study.

Participants were recruited from three universities in Jiangxi Province, China, utilizing a convenience sampling strategy. We acknowledge that convenience sampling is prone to selection bias and may limit the generalizability of the findings. However, this approach was deemed appropriate given the exploratory nature of the study and financial constraints. To enhance the representativeness of the sample, we selected three large public universities located in Nanchang, the provincial capital. These universities are comprehensive institutions situated in an urban area, offering a wide range of academic programs from which participants were drawn, thus capturing a diverse student body that, to some extent, reflects the broader population of female university students in urban China. Data collection began on March 20, 2025, and lasted for 1 month. The survey was distributed through an online questionnaire^1^ and shared via student organizations, and social media platforms, such as WeChat and QQ, resulting in an initial 832 responses. Initially, The following criteria must be met in order to participate in this study: (1) The participant must be a full-time university student; (2) Participants must be biologically female; (3) The participant must have no history of mental illness; (4) Participants must not have participated in similar studies; (5) The participant must voluntarily agree to participate and sign an informed consent form. Subsequently, a multi-stage data screening plan will be adopted to ensure the quality and integrity of the final data set. First, to prevent duplicate entries, the survey platform was configured to permit only one submission per unique IP address. Following collection, each case was reviewed, and responses were invalidated and removed if they were completed in an implausibly short duration or displayed invariant response patterns (i.e., “straight-lining”) across any scale. Next, to ensure the normality of the data, we excluded data points with absolute skewness greater than 1 and kurtosis greater than 3 in accordance with common statistical practices. Through this comprehensive screening process, a total of 43 responses were excluded. This resulted in a final valid sample of 789 participants being retained for statistical analysis, corresponding to a valid response rate of 94.8%. The design of the online instrument mandated responses for all items, thus eliminating any missing data in the final dataset.

The average age of our sample was 20.49 (SD = 1.929). Participants received monetary compensation of 5 CNY (approximately US$0.70) via electronic transfer for their participation. Before testing, the participants were told that the study was voluntary and anonymous, that their information would be kept secret, and that they could stop at any time. Our institution’s ethics committee approved this study.

### 3.2 Measures

#### 3.2.1 Negative emotion

This study employed the Depression Anxiety Stress Scale (DASS) developed by Lovibond and Lovibond (77), a tool that has been demonstrated to possess adequate reliability and validity among Chinese adolescents and students (78, 79). The scale consists of 21 items (e.g., “I found it difficult to relax”), employing a 4-point Likert scale (0 = “Did not apply to me at all” to 3 = “Applied to me very much or most of the time”). Higher total scores indicate elevated levels of NE. The internal consistency reliability was satisfactory, with a Cronbach’s *α* of 0.879.

#### 3.2.2 Eating behavior

This study employed the abbreviated version of the Sakata Eating Behavior Scale short form, which was developed by Tayama et al. (80) and revised into Chinese by Ge et al. (81), and demonstrated adequate reliability and validity. The scale consists of seven items (e.g., “Like oily foods”). A 4-point Likert scale was used, ranging from 1 (strongly disagree) to 4 (strongly agree). High scores indicate a greater prevalence of problematic EB. The internal consistency reliability was satisfactory, with a Cronbach’s α of 0.874.

#### 3.2.3 Physical exercise

This study assessed participants’ PE through a single item: “Over the past 7 days, how many days did you engage in at least 20 min of PE or activity that made you sweat or breathe heavily?” In all analyses, the physical exercise (PE) variable, measured as the number of days of activity per week (ranging from 0 to 7), was treated as a continuous variable. This measurement method, while not capturing the full complexity of exercise duration or intensity, has been shown to be a practical and reasonably valid indicator of physical activity levels in large-scale survey research (82). It has been validated in previous health behavior studies (83, 84).

### 3.3 Data analysis

All statistical analyses were conducted using SPSS 27.0 and Mplus 8.3, with the significance level set at α = 0.05. The analysis proceeded in three stages. First, descriptive statistics for all study variables were computed, and Pearson correlation analyses were performed to examine their bivariate relationships. Second, the hypothesized mediating effect of PE was tested using Model 4 of the PROCESS macro (v.3.5) for SPSS (85). Furthermore, Body Mass Index (BMI) was included as a Control variable in the model. The indirect effect was considered statistically significant if the 95% confidence interval, generated from 5,000 bootstrap samples, did not contain zero. Third, to identify heterogeneous subgroups based on NE, LPA was conducted in Mplus 8.3 using the three dimensions of the DASS scale as indicators, testing models with 1–5 classes. The optimal model was selected based on a comprehensive evaluation of multiple fit indices (86): the Akaike Information Criterion (AIC), Bayesian Information Criterion (BIC), and sample-size adjusted BIC (aBIC), where lower values indicate better fit; Entropy, where values approaching 1.0 reflect higher classification accuracy; and the Lo–Mendell–Rubin adjusted likelihood ratio test (LMR-LRT) and Bootstrap Likelihood Ratio Test (BLRT), where a significant *p*-value suggests that the k-class model is a significant improvement over the k-1 class model. Following the identification of the optimal latent profile, one-way analysis of variance and post-hoc tests were used to examine differences in eating behavior and physical exercise across the NE latent profiles.

## 4 Results

### 4.1 Demographic information on female college students

The female college students are predominantly undergraduates (88.30%), with 10.80% master’s and 0.90% doctoral students. Most have a normal BMI (46.50%), while 30.40% are underweight, 21.30% overweight, and 1.80% obese. The results of the analysis of variance revealed that there were no statistically significant differences between groups in terms of education among female college students in NE (*F* = 0.509), EB (*F* = 0.386), and PE (*F* = 2.682). Conversely, statistically significant differences were observed among the groups in terms of BMI among female college students in NE (*F* = 11.532, *p* < 0.001), EB (*F* = 4.378, *p* < 0.01), and PE (*F* = 11.532, *p* < 0.01). Table 1 shows the demographic information of the sample.

**Table 1:** Basic demographic characteristics of participants.

	*n*(%)	NE	EB	PE
Education		0.509	0.386	2.682
Undergraduate	697 (88.30%)			
Master’s student	85 (10.80%)			
Doctoral student	7 (0.90%)			
BMI		11.532^***^	4.378^**^	4.099^**^
<18.5	239 (30.40%)			
18.5–24.9	366 (46.50%)			
25–29.9	168 (21.30%)			
≥30	14 (1.80%)			

ANOVA results (two-tailed test). *p < 0.05, **p < 0.01, ***p < 0.001.

### 4.2 Correlation analysis

Table 2 presents means, standard deviations, skewness, kurtosis, and correlations among NE, EB, and PE. Results revealed significant positive correlations between NE and EB, while PE was negatively correlated with both NE and EB.

**Table 2:** Correlation coefficients of negative emotion, eating behavior, and physical exercise in female college students.

	M	SD	Skewness	Kurtosis	NE	EB	PE
NE	1.473	0.588	−0.349	−0.072	1		
EB	2.652	0.932	0.774	−0.056	0.458**	1	
PE	3.380	2.099	−0.007	−1.097	−0.400**	−0.259**	1

NE, Negative Emotion; EB, Eating Behavior; PE, Physical Exercise. **p < 0.01. M, mean. SD, standard deviation.

### 4.3 The mediation analyses

**Figure 1:**
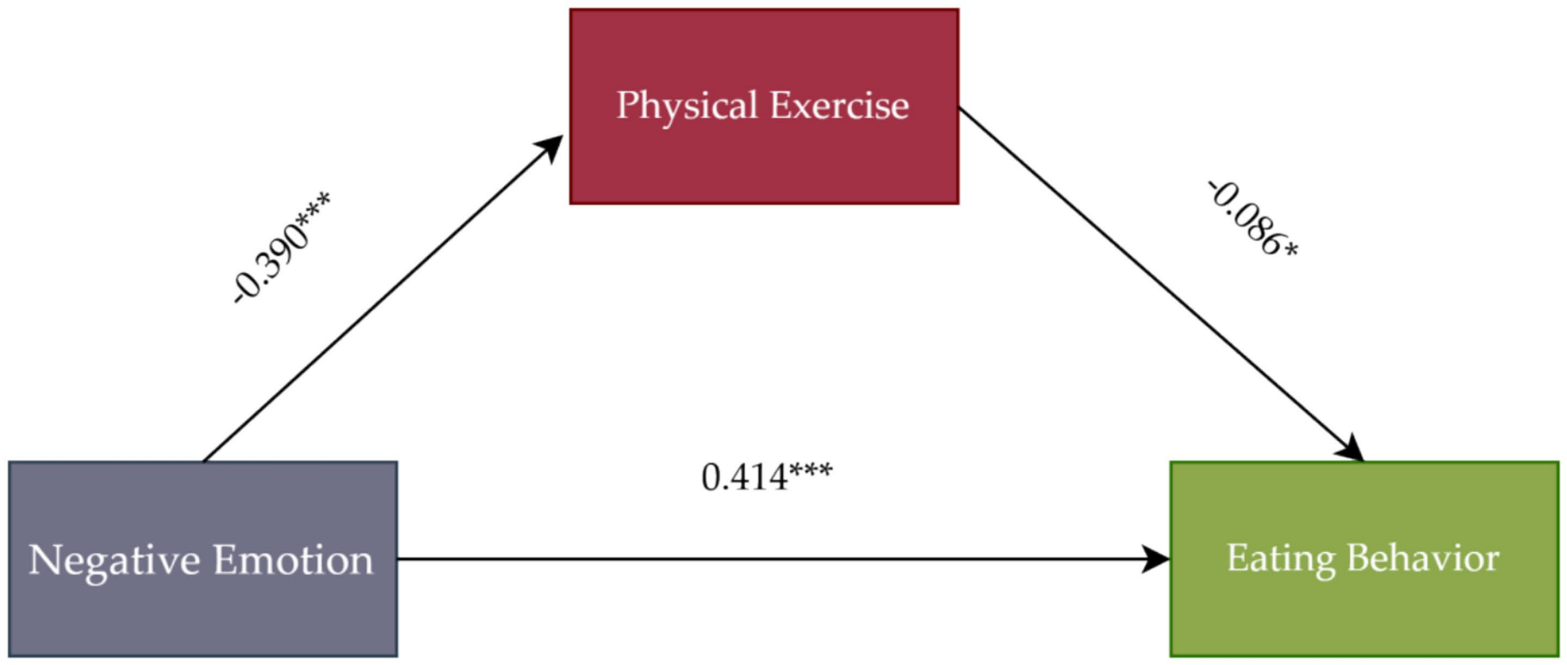
Mediation effect diagram.

Variance Inflation Factor was computed to assess multicollinearity among variables. The highest variance inflation factor value was 1.190, below the threshold of 5, indicating no substantial multicollinearity issues. After standardizing the data and controlling for BMI, NE positively affected EB (*β* = 0.414, *t* = 11.889, *p* < 0.001) and negatively affected PE (*β* = −0.390, *t* = −11.730, *p* < 0.001). Additionally, PE significantly affected EB (*β* = −0.086, *t* = −2.506, *p* < 0.05), with the 95% confidence interval not containing zero (Table 3). The indirect effect of PE (0.034) accounted for 8% of the total effect (0.448). While the proportion of the mediation effect is modest, its statistical significance suggests that physical exercise is a meaningful, albeit small, pathway through which negative emotions are linked to eating behaviors in this population. Mediation analysis results (Table 4) indicated that PE mediated the relationship between NE and EB, supporting Hypothesis 1 (Figure 1). Together, NE and PE explained 21.6% of the variance in EB, further confirming their significant predictive power over eating behavior.

**Table 3:** Regression analyses of the effects of negative emotion and physical exercise on eating behavior.

Variables	Model1		Model2		Model3	
	(PE)		(EB)		(EB)	
	*β*	*t*	*β*	*t*	*β*	*t*
NE	−0.390	11.730^***^	0.414	11.889^***^	0.448	13.893^***^
PE			−0.086	−2.506^*^		
BMI	−0.052		0.051	1.204	0.005	1.309
R^2^	0.160		0.216		0.210	
*F*	74.665^***^		72.064***		104.254^***^	

*p < 0.05, **p < 0.01, ***p < 0.001.

**Table 4:** Mediating effects of physical exercise between negative emotion and eating behavior.

	Effect	Boost se	95%CI		Relativistic effect
			LLCI	ULCI	
Total	0.448	0.032	0.384	0.511	
NE → EB	0.414	0.035	0.346	0.482	92%
NE → PE → EB	0.034	0.011	0.012	0.056	8%

NE, Negative Emotion; EB, Eating Behavior; PE, Physical Exercise.

### 4.4 Latent profile analyses

As shown in Table 5, the class3 model demonstrated the highest entropy value (0.895), indicating clear class distinction. This model also yielded relatively lower AIC, BIC, and aBIC values among all candidate models, suggesting excellent balance between goodness of fit and model complexity. Furthermore, both LMR and BLRT tests were significant (*p* < 0.001). Based on these criteria, the class3 model was identified as the optimal fitting model.

**Table 5:** Comparison of fit indices for latent profile analysis models.

Model	LL	AIC	BIC	aBIC	LMR(*P*)	BLRT(*P*)	Entropy	Categorical probability%
Class 1	−2698.983	5409.966	5437.991	5418.938				
Class 2	−2510.195	5040.390	5087.097	5055.342	<0.0001	<0.0001	0.861	17.24%/82.76%
Class 3	−2410.740	4849.480	4914.871	4870.413	<0.0001	<0.0001	0.895	16.86%/ 51.33%/31.81%
Class 4	−2366.791	4769.582	4853.656	4796.497	<0.001	<0.0001	0.882	6.34%/16.86%/ 24.33%/52.47%
Class 5	−2335.462	4714.925	4817.682	4747.820	<0.0001	<0.0001	0.886	3.68%/ 13.44%/53.23%/ 13.43%/16.22%

**Figure 2:**
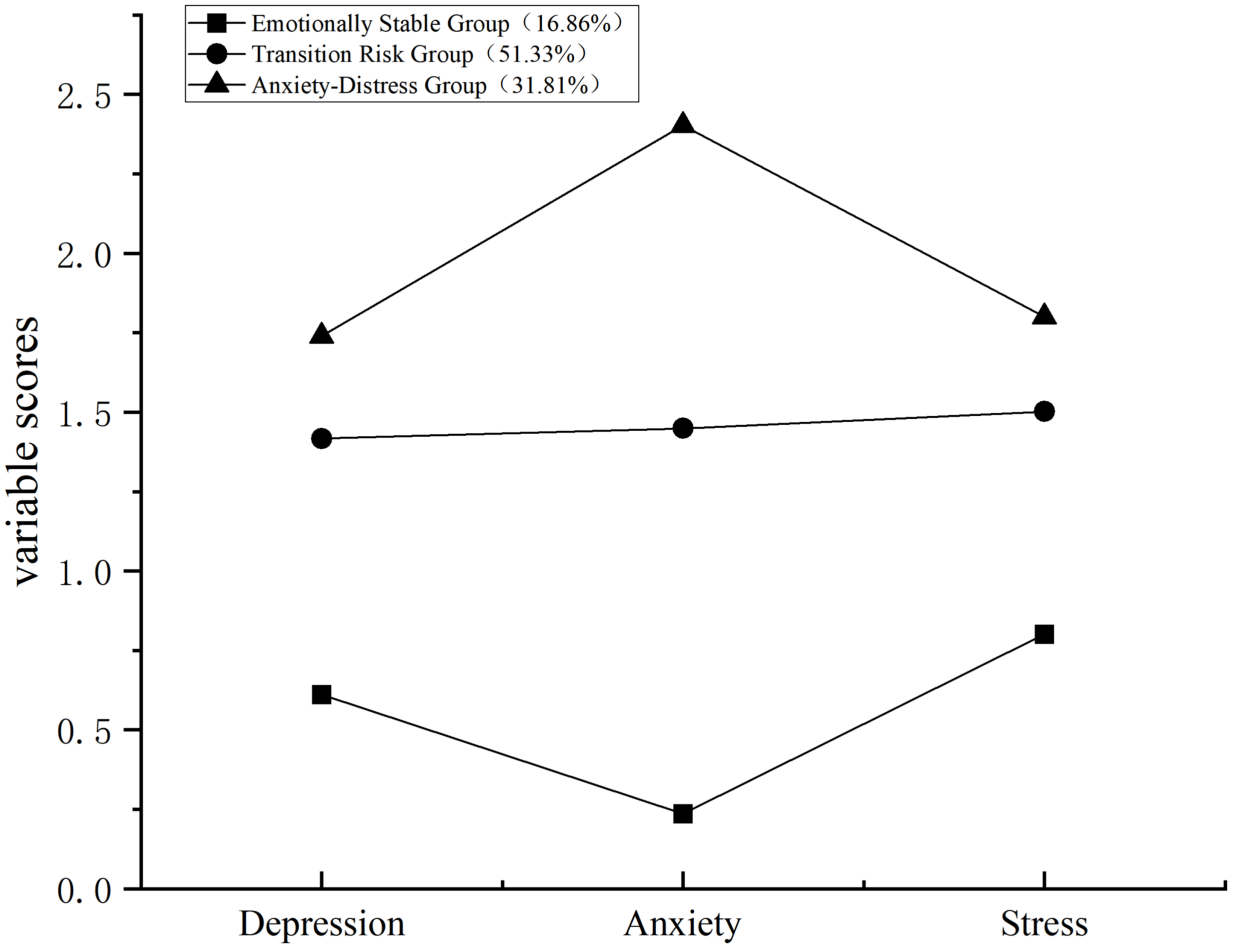
Latent classes of negative emotion.

LPA identified three categories of NE among female university students. As shown in Figure 2, significant differences emerged across all dimensions. Based on these characteristic patterns, we designated the categories as: Emotionally Stable Group (C1, 16.86%), Transition Risk Group (C2, 51.33%), and Anxiety-Distress Group (C3, 31.81%), supporting Hypothesis 2.

### 4.5 Effect of latent profile classification

As shown in Table 6, significant differences were found among female college students with different NE profiles in both EB [*F* (2,786) = 65.088, *p* < 0.001] and PE [F (2,786) = 55.241, *p* < 0.001]. *Post-hoc* analyses revealed that EB scores increased progressively from the Emotionally Stable Group to the Transition Risk Group to the Anxiety-Distress Group, while PE scores showed the opposite pattern. These findings demonstrate significant differences in EB and PE across different latent profiles of negative affect, supporting Hypothesis 3.

**Table 6:** Descriptive statistics and group differences in EB and PE across latent profiles (M ± SD).

Variable	C1(133)	C2s(405)	C3(251)	*F*	Back texting
EB	2.034 ± 0.952	2.591 ± 0.654	3.076 ± 1.087	65.088^***^	C3 > C2 > C1
PE	4.790 ± 1.200	3.410 ± 2.221	2.570 ± 1.856	55.241^***^	C1 > C2 > C3

C1, Emotionally Stable Group; C2, Transition Risk Group; C3, Anxious-Distress Group. ***p < 0.001.

## 5 Discussion

This study employed LPA to investigate the interrelationships among NE, EB, and PE in female college students. Specifically, we examined how heterogeneous patterns of NE relate to EB, with PE serving as a potential mediator. Our findings revealed that PE significantly mediated the relationship between NE and EB. Furthermore, we identified three distinct latent profiles of NA among female college students, each associated with EB and PE. These results align with cognitive-behavioral theory.

### 5.1 The mediating effect of physical exercise

The results of this study indicate that PE plays a significant mediating role between NE and EB among female college students, which aligns with our research hypothesis. Previous studies have found a strong association between NE and PE (87), and the positive effects of PE on improving EB have also been well-documented (88). The contribution of this study lies in connecting these two areas of research, revealing that NE may not only directly drive individuals to emotional eating (89) but also indirectly reinforce unhealthy eating patterns by ‘depriving’ individuals of the motivation and behavior to engage in PE as a healthy coping mechanism (90). According to cognitive-behavioral theory, individuals in a negative emotional state often have their cognitive systems dominated by automated negative thoughts (91), such as “I’m too tired.” These cognitive factors directly lead to abandoning activities like PE that require willpower and energy (92). This behavioral inaction, in turn, deprives individuals of the opportunity to gain a sense of accomplishment and positive emotional experiences from PE, thereby trapping EB and low PE participation in a vicious cycle. In the context of this study, when female college students experience NE, they may be influenced by these automated negative thoughts, thereby reducing the likelihood of engaging in PE, which in turn increases the probability of emotional eating behavior. At the neurobiological level, studies suggest that PE can improve the function of the brain’s dopamine pathway and increase the circulation of neurotrophins (e.g., VEGF), thereby physiologically reducing an individual’s emotional eating (93). From the perspective of self-regulation theory, PE can also be viewed as a self-regulatory behavior. Self-regulation theory emphasizes that individuals manage their health and emotions by setting goals, monitoring their own behavior and emotional states, and adjusting their behavior according to their goals (94). Students who are able to stick with physical exercise tend to show stronger self-discipline and delayed gratification (95). This self-regulation ability itself encourages individuals to resist emotional impulses (such as emotional eating) and regulate their own behavior (96). In this study, PE may not only be an immediate coping strategy for negative emotions but also a self-regulatory behavior that female college students adopt to manage their emotions and health in the long term. Therefore, it is suggested that universities could launch intervention programs combining physical exercise with emotional management for female college students, helping them establish positive exercise habits and enhance their self-regulation abilities to better cope with negative emotions and reduce the occurrence of emotional eating behavior.

### 5.2 Latent profile of negative emotions

This study employed LPA to investigate the latent profile structure of NE among female university students. Based on a comprehensive assessment of multiple model fit indices (e.g., AIC, BIC, and aBIC), a three-profile model was ultimately identified as optimal. This finding supports Hypothesis 2 and is consistent with previous research identifying distinct mental health subgroups in similar populations (97). We named these three profiles based on their scoring patterns across the DASS subscales: the Emotionally Stable group, the Transition Risk group, and the Anxious-Distress group. The Emotionally Stable group (accounting for 16.9% of the total sample) was characterized by consistently low scores on all negative emotion indicators, demonstrating a positive and healthy psychological state. In contrast, the Anxious-Distress group (31.8%) exhibited high levels of anxiety and moderately high levels of depression and stress, representing the highest-risk profile. Notably, the most prevalent category was the ‘Transition Risk’ group, comprising a majority of the sample at 51.3%. This group was distinguished by moderately elevated scores across the dimensions, with particular prominence in stress levels. This finding reveals the existence of a large “intermediate” subgroup among female university students. While not in acute distress, their heightened stress levels suggest a vulnerability, making them a core target for mental health prevention and early intervention efforts. These findings have direct implications for intervention. To enhance the applied significance of our findings, we propose a tiered approach to intervention based on the identified emotional profiles. For the high-risk “Anxious-Distress” group, intensive, one-on-one interventions like cognitive-behavioral therapy (CBT) or professional counseling are necessary to address their significant psychological distress. For the large “Transition Risk” group, broader, skill-based group interventions would be most effective. For instance, universities could offer workshops on mindfulness-based stress reduction or organize accessible and enjoyable group fitness classes (e.g., yoga, dance, or team sports) that foster social connection and reduce barriers to participation. For the “Emotionally Stable” group, the focus should be on health promotion and reinforcing existing positive behaviors through campus-wide wellness campaigns. In summary, this three-profile model transcends the traditional variable-centered perspective, providing a more refined classification framework for precisely identifying female college students at different risk levels.

### 5.3 Effect of latent profile classification on physical activity and eating behavior

A central finding of this study, which strongly supports Hypothesis 3, is that the three latent profiles of negative emotion are significantly associated with EB and PE. Post-hoc analyses revealed a clear gradient effect: the Anxious-Distress group reported the most problematic EB and the lowest levels of PE, followed by the Transition Risk group, with the Emotionally Stable group exhibiting the healthiest behaviors. This demonstrates unequivocally that the severity of NE is a key predictor of these health-related outcomes. The finding that the Anxious-Distress group exhibited the most severe behavioral problems aligns with the Emotion Dysregulation Model (98). This model posits that when individuals face pervasive and intense negative affect, they may resort to maladaptive behaviors like emotional eating or physical inactivity to numb or escape from unbearable internal distress (99, 100). For this high-risk group, unhealthy eating and exercise avoidance are likely not isolated choices but rather functional, albeit detrimental, responses to a state of comprehensive psychological dysregulation. Notably, the “Transition Risk” group, which comprised the largest proportion of the sample (51.33%), warrants special attention. While not in acute distress, their moderately elevated scores, particularly in stress, suggest they represent a psychologically vulnerable population. This group may be at a critical juncture, at risk of developing more severe maladaptive behaviors if faced with additional stressors. Interventions for this group should therefore focus on prevention and building resilience. Practical examples include university-led workshops on stress management and coping skills, establishing peer support networks, and promoting accessible campus-based physical activities to buffer against the negative impact of stress. Finally, the Emotionally Stable group, being least affected by NE, serves as a healthy baseline, confirming that positive emotions are foundational to maintaining positive health behaviors (101). In summary, by employing a person-centered approach, this study not only identifies which students are at risk but also reveals how their specific emotional profiles translate into distinct behavioral patterns. This study provides empirical evidence for designing targeted, tiered interventions for female university students in the future.

### 5.4 Limitations

This study has several limitations that should be acknowledged. First, the cross-sectional nature of this study inherently prevents definitive causal inferences regarding the observed relationships. Specifically, we cannot ascertain whether negative emotions precipitate reduced physical activity engagement or whether physical inactivity exacerbates negative emotional states—a bidirectional ambiguity that warrants clarification. While our current investigation focused on physical exercise (PE) as the primary mediator, future studies should expand this scope by incorporating additional mediating pathways such as family atmosphere and self-regulation. Furthermore, extending LPA of NE to diverse populations (e.g., clinical cohorts, adolescents) would enhance generalizability. To address these limitations and advance causal understanding, we strongly endorse implementing longitudinal or experimental designs in subsequent research.

Second, all data were collected via self-report measures, which may be subject to social desirability bias and recall errors. Although we implemented measures to ensure anonymity and encourage honest responses, the potential for such biases cannot be entirely eliminated.

Third, a methodological limitation is the use of a single-item measure for PE. This approach is insufficient to comprehensively capture exercise behavior, as it does not account for the duration, intensity, or type of physical activity. This may have led to an underestimation of the true relationship between PE and the other variables. Future research should incorporate more robust, multi-dimensional assessments of physical activity, or even objective measures like accelerometers.

In Addition, a limitation is the lack of screening for several key confounding variables. We did not collect data on whether participants had been formally diagnosed with eating disorders (e.g., anorexia or bulimia nervosa). Furthermore, other crucial lifestyle and social factors, such as sleep patterns, specific dietary regimens (e.g., medical or self-imposed diets), and levels of social support, were not assessed. These factors could substantially influence the core variables of our study—NE and EB—thereby potentially confounding the observed relationships. Consequently, our findings should be interpreted with caution, as the presence of these unmeasured variables may limit the internal validity of our conclusions. Future studies should incorporate specific screening questions to control for these confounders.

Finally, since the study subjects were conveniently sampled from only three public universities in Jiangxi Province, China, the generalizability of the study results to female college students nationwide and internationally is limited. Jiangxi province, for instance, has unique economic and cultural characteristics compared to other areas, which may result in different academic pressures, social norms, and lifestyle patterns. Therefore, the prevalence of certain emotional profiles and their association with eating and exercise behaviors might differ in other regions. Furthermore, this study did not compare the demographic characteristics of female college students, such as their academic backgrounds, across multiple groups. Future studies should focus on recruiting more diverse and representative samples and using multiple methods to validate these findings.

## 6 Conclusion

This study identified three distinct emotional profiles among female university students—“Emotionally Stable,” “Transition Risk”, and “Anxious-Distress”—and confirmed that physical exercise partially mediates the link between negative emotions and eating behaviors. These findings carry significant practical implications for university mental health programs. Instead of a one-size-fits-all approach, institutions should adopt a tiered, profile-based intervention model. For instance, high-risk students require intensive counseling, whereas the large at-risk group would benefit from preventative workshops on stress management and resilience. For low-risk students, general health promotion can reinforce their well-being. By tailoring interventions to students’ specific emotional needs, universities can more effectively foster psychological resilience and promote healthier lifestyles. These conclusions should be considered in light of the study’s cross-sectional design and its reliance on self-report measures.

## Data Availability

The original contributions presented in the study are included in the article/Supplementary material, further inquiries can be directed to the corresponding author.

## Footnotes

^1^ www.wjx.cn
