# Supplementary material for: The latent profile structure of negative emotion in female college students and its impact on eating behavior: the mediating role of physical exercise
Source: Front Public Health. 2025 Aug 13;13:1663474. doi: 10.3389/fpubh.2025.1663474 (PMC12380551; doi:10.3389/fpubh.2025.1663474)
Supplement: Supplementary file 1 [file Supplementary_file_1.docx]

**Sakata Eating Behavior Scale Short Form**

Scoring method: This scale is used to assess female college students' eating behaviors and consists of seven items. A four-point Likert scale is used, with scores ranging from 1 (strongly disagree) to 4 (strongly agree). The higher the total self-assessment score, the higher the incidence of problematic eating behaviors.

| No. | Question Entry | strongly disagree | somewhat disagree | somewhat agree | strongly agree |
| --- | --- | --- | --- | --- | --- |
| 1 | Eat at all different times. | 1 | 2 | 3 | 4 |
| 2 | Do not feel satisfied unless I eat until full. | 1 | 2 | 3 | 4 |
| 3 | Eat fast. | 1 | 2 | 3 | 4 |
| 4 | Tend to gain weight more easily than others. | 1 | 2 | 3 | 4 |
| 5 | Like oily foods. | 1 | 2 | 3 | 4 |
| 6 | Eat if others around me are eating. | 1 | 2 | 3 | 4 |
| 7 | When buying food, I am not content unless I buy more than necessary. | 1 | 2 | 3 | 4 |
