# Supplementary material for: The latent profile structure of negative emotion in female college students and its impact on eating behavior: the mediating role of physical exercise
Source: Front Public Health. 2025 Aug 13;13:1663474. doi: 10.3389/fpubh.2025.1663474 (PMC12380551; doi:10.3389/fpubh.2025.1663474)
Supplement: Supplementary file 2 [file Supplementary_file_2.docx]

**Depression Anxiety Stress Scale**

Scoring method: This scale is used to assess negative emotions in female college students. It consists of 21 items and uses a 4-point Likert scale (0 = “completely disagree” to 3 = “strongly agree or agree most of the time”). The higher the total self-assessment score, the higher the level of negative emotions.

| No. | Question Entry | Did not apply to me at all | Applied to me to some degree, or some of the time | Applied to me to a considerable degree or a good part of time | Applied to me very much or most of the time |
| --- | --- | --- | --- | --- | --- |
| 1 | (s）I found it hard to wind down | 0 | 1 | 2 | 3 |
| 2 | (s) I tended to over-react to situations 0 1 2 3 | 0 | 1 | 2 | 3 |
| 3 | (s) I felt that I was using a lot of nervous energy | 0 | 1 | 2 | 3 |
| 4 | (s) I found myself getting agitated | 0 | 1 | 2 | 3 |
| 5 | (s) I found it difficult to relax | 0 | 1 | 2 | 3 |
| 6 | (s) I was intolerant of anything that kept me from getting on with what I was doing | 0 | 1 | 2 | 3 |
| 7 | (s)I felt that I was rather touchy | 0 | 1 | 2 | 3 |
| 8 | (a)I was aware of dryness of my mouth | 0 | 1 | 2 | 3 |
| 9 | (a) I experienced breathing difficulty (e.g. excessively rapid breathing, breathlessness in the absence of physical exertion) | 0 | 1 | 2 | 3 |
| 10 | (a) I experienced trembling (e.g. in the hands) | 0 | 1 | 2 | 3 |
| 11 | (a) I was worried about situations in which I might panic and make a fool of myself | 0 | 1 | 2 | 3 |
| 12 | (a) I felt I was close to panic | 0 | 1 | 2 | 3 |
| 13 | (a) I was aware of the action of my heart in the absence of physical exertion (e.g. sense of heart rate increase, heart missing a beat) | 0 | 1 | 2 | 3 |
| 14 | (a) I felt scared without any good reason | 0 | 1 | 2 | 3 |
| 15 | (d) I couldn’t seem to experience any positive feeling at all | 0 | 1 | 2 | 3 |
| 16 | (d) I found it difficult to work up the initiative to do things | 0 | 1 | 2 | 3 |
| 17 | (d) I felt that I had nothing to look forward to | 0 | 1 | 2 | 3 |
| 18 | (d) I felt down-hearted and blue | 0 | 1 | 2 | 3 |
| 19 | (d) I was unable to become enthusiastic about anything | 0 | 1 | 2 | 3 |
| 20 | (d) I felt I wasn’t worth much as a person | 0 | 1 | 2 | 3 |
| 21 | (d) I felt that life was meaningless | 0 | 1 | 2 | 3 |
